# A Complex Life Habitable Zone Based On Lipid Solubility Theory

**DOI:** 10.1038/s41598-020-64436-z

**Published:** 2020-05-04

**Authors:** Ramses M. Ramirez

**Affiliations:** 1 Earth-Life Science Institute, Tokyo Institute of Technology, Tokyo, Japan; 2 Space Science Institute, Boulder, Co USA

**Keywords:** Astrobiology, Exoplanets, Astronomy and planetary science, Respiration

## Abstract

To find potentially habitable exoplanets, space missions employ the habitable zone (HZ), which is the region around a star (or multiple stars) where standing bodies of water could exist on the surface of a rocky planet. Follow-up atmospheric characterization could yield biosignatures signifying life. Although most iterations of the HZ are agnostic regarding the nature of such life, a recent study argues that a complex life HZ would be considerably smaller than that used in classical definitions. Here, I use an advanced energy balance model to show that such an HZ would be considerably wider than originally predicted given revised CO_2_ limits and (for the first time) N_2_ respiration limits for complex life. The width of this complex life HZ (CLHZ) increases by ~35% from ~0.95–1.2 AU to 0.95–1.31 AU in our solar system. Similar extensions are shown for stars with stellar effective temperatures between 2,600–9,000 K. I define this CLHZ using lipid solubility theory, diving data, and results from animal laboratory experiments. I also discuss implications for biosignatures and technosignatures. Finally, I discuss the applicability of  the CLHZ and other HZ variants to the search for both simple and complex life.

## Introduction

The habitable zone (HZ) is the region around a star (or multiple stars) where standing bodies of liquid water could exist on the surface of a rocky planet^1–3^. To date, the HZ remains the principle means by which potentially habitable planets are targeted for follow up spectroscopic observations^2^.

In the classical HZ definition of Kasting *et al*.^1^, the interplay of CO_2_ and H_2_O determines the habitability of planets over an inferred carbonate-silicate cycle that operates over the main-sequence phase of stellar evolution^1^. However, the HZ is an atmospheric composition dependent concept that is highly sensitive to the assumptions made^4^. Alternative definitions include extensions involving hydrogen^5,6^, methane^7^, or applications to the pre-main-sequence or post-main-sequence phases of stellar evolution^8–10^. Thus, an ongoing debate exists regarding which HZ to use and under which circumstances^2,11–13^.

Although some core assumptions may differ among HZ definitions, all inherently assume that both simple and complex life are searched and make no distinction between the two. Indeed, much of the classical HZ supports atmospheric conditions that are hostile to Earth-like animal and plant life^1^.

Recently, Schwieterman *et al*.^14^ used a 1-D radiative-convective climate model to argue that a HZ for complex life (e.g. animals and plants) would be significantly narrower than the classical main-sequence CO_2_-H_2_O HZ^1,3^. They correctly argue that the CO_2_ pressures that human and animal life can adapt to would be lower than those inferred for other HZ definitions. Their calculated complex life HZ outer edge spans a wide range of CO_2_ pressures (0.01 bar to 1 bar) that correspond to a similarly unconstrained range in orbital distances (~1.1–1.3 AU in our solar system). However, a tighter HZ definition is useful in the astronomical search for potentially habitable planets. Here, we apply insights from laboratory experiments and lipid solubility theory^15–17^ to more tightly constrain CO_2_ and (for the first time) *N*_2_ atmospheric pressure limits for Earth-like complex life. Incorporating these updated constraints, I use an advanced latitudinally-dependent energy balance model with clouds to show that such an HZ is significantly wider than previously estimated^14^. I caveat this analysis by acknowledging that such terrestrial N_2_ and CO_2_ limits may or may not apply to other planets. This is because we do not know the capacity of complex alien life (with a different evolutionary lineage) to adapt to very high (multi-bar) N_2_ or CO_2_ pressures. Only future observations and studies can address this point. Nevertheless, astrobiology research often assumes that certain characteristics common to life on Earth (e.g., carbon-based life, the abundance of water) may be universal. Similarly, the hypothesis that extraterrestrial complex life may be bound by similar respiratory constraints as is life on Earth makes this Complex Life HZ (CLHZ) potentially useful in the astronomical search for life^14,18,19^.

It is often incorrectly assumed that complex life only refers to metazoans and protists. However, plants and many fungi are also very complex. Nevertheless, little work has systematically assessed the survivability of such organisms to the elevated atmospheric CO_2_ or N_2_ pressures considered here. Thus, the criteria necessarily limit such complex life to metazoan animals, including humans, that directly breathe atmospheric air. Animals that breath underwater using gills (like fish and squid) are not included for this reason (although such marine animals may be bound by similar aquatic CO_2_ limits^14^). Cetaceans (e.g., whales and dolphins) satisfy the criteria because they have lungs and must resurface for air. Using this framework, I discuss CO_2_ and N_2_ respiratory limits for humans and other air-breathing animals based on our current understanding of complex life on Earth. In extrapolating these concepts to the CLHZ, I assume that complex life elsewhere is bound by similar N_2_ and CO_2_ respiratory limits as are humans and other terrestrial air-breathing animals (discussed more in Supplementary Information).

## CO_2_ and N_2_ Limits For Complex Life Habitable Zone

Centers for Disease Control (CDC) guidelines find that atmospheric CO_2_ concentrations exceeding 40,000 ppm (0.04 bar in a 1 bar atmosphere) are immediately dangerous to human life. This CDC assessment is based on quick and harmful exposure (acute) inhalation data in humans^20^. Common symptoms include dizziness, headache, and shortness of breath, which are similar to those of altitude sickness caused by low O_2_ (hypoxia). The negative effects of acute hypoxia can occur at elevations as low as 1,500 m. Indeed, rapid ascent to heights above ~2,500 m can trigger pulmonary edema and death in otherwise healthy individuals^21^. Even so, humans have proven capable of adapting to and even thriving at much higher altitudes. La Paz (population 2.7 million, ~0.12 bar O_2_), the capital of Bolivia, rests at an elevation above 3,650 m and La Rinconada (population 30,000), the highest permanent settlement in the world, has an elevation of 5,100 m^22^. Thus, what such CDC guidelines for CO_2_ exposure do not factor is the ability of humans (and other animals) to gradually acclimate to non-standard conditions. I attempt to estimate that component here.

Although very high CO_2_ acclimatization experiments would be dangerous to perform on humans, members of our species have successfully adapted to CO_2_ pressures of 0.04 bar^23^. Multiple studies have assessed how animals acclimate to even higher CO_2_ doses. For instance, rhesus monkeys have been acclimated to 0.06 bar CO_2_ to no ill effect^24^. Sheep exhibited some minor discomfort at 0.08 bar, but it was tolerable until 0.12 bar^25,26^, with death not occurring until 0.16 bar^25,26^. It is worth noting that an acclimatization threshold was only achieved in the sheep experiment, but not with the rhesus monkeys. Such experiments suggest a CO_2_ limit for animals of at least 0.1 bar (an expanded summary of laboratory results is found in Supplementary Information), but can this be estimated theoretically? And, in turn, can similar respiration limits be determined for N_2_?

**Figure 1:**
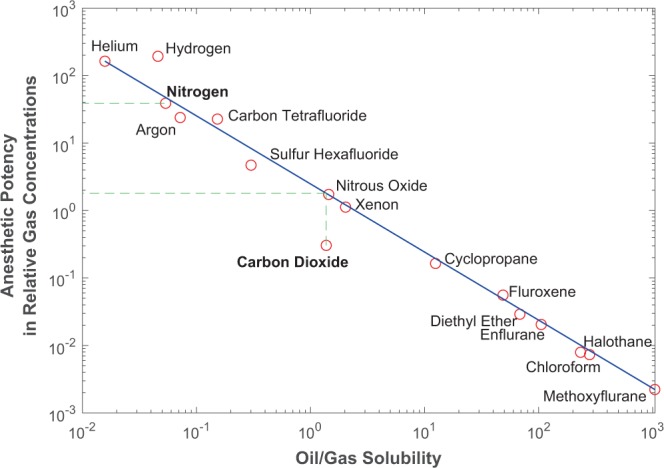
The Meyer-Overton correlation^15–17,29^, of oil/gas solubility versus anesthetic potential of inhaled gases. Figure recreated from published data^15–17,29^.

There are three important effects to consider in determining how much CO_2_ our bodies can withstand. The first is that CO_2_ could replace O_2_ in the blood, which deprives oxygen. However, this inhibition of O_2_ hemoglobin effect is negligible because bicarbonates, not blood hemoglobin, transport most of the CO_2_ in the blood^27^. The second effect, respiratory acidosis, is the reduction of blood pH at higher CO_2_ levels and is the major cause of CO_2_ toxicity. However, our kidneys can neutralize the carbonic acid formed in the blood (in a process called renal compensation), allowing acclimatization to proceed^28^. Eventually, once gas pressures become too high, even kidney pH neutralization becomes insufficient to counter such an ill response. It is this effect, respiratory narcosis, that causes further breathing of the gas to produce symptoms similar to intoxication as the gas become anesthetic. This respiratory narcosis effect in our blood is related to its solubility in lipids and oils by the Meyer-Overton correlation^15–17^ (Fig. 1).

According to Fig. 1, most gases plot along a straight line. Chloroform is a great anesthetic because it can induce intoxication at relatively low pressures whereas helium requires much higher pressures to incur the same anesthetic effects. CO_2_ is much more narcotic than what lipid-solubility theory predicts, but that is due to respiratory acidosis, which can be overcome by renal compensation. Thus, the true narcotic potential of CO_2_ is most similar to that of N_2_O, which is ~21 times more narcotic than N_2_ (Fig. 1), consistent with ref. ^29^. For N_2_, the recommended safety limits for deep divers above which serious intoxication occurs is equivalent to 4 bar atmospheric pressure at 30 m^29^ (1 additional bar for each 10 m below sea level) or ~3.1 bar N_2_ in an Earth-like (78% N_2_) atmosphere, assuming that O_2_ has a similar narcotic toxicity as N_2_. This deep diving narcosis occurs because the air that divers breathe at depth becomes more compressed at increasingly higher pressures. In contrast, if renal compensation were ignored, the respiratory narcosis limit would be (3.1/100) ~ 0.03 bar, much too low relative to the above experiments. This again shows why the effects of renal compensation must be considered. Thus, the correct respiratory narcosis limit for CO_2_ is (3.1/21) ~0.15 bar, which is consistent with the animal experiments. However, laboratory experiments with rats suggest a slightly lower CO_2_ tolerance for newborns than for adults, closer to 0.1 bar^30^. In the absence of N_2_ respiration data for newborns, I assume that newborns would have a lower threshold tolerance for N_2_ as well, approximately 2/3 that of the theoretical adult limit (2 bar).

As per classical HZ calculations, the current simulated atmospheres are dominated by two atmospheric gases, N_2_ and CO_2_. Given the above theoretical and experimental considerations, I define the outermost extent of the CLHZ by the distance where equatorial temperatures can exceed the freezing point of water (273 K), assuming atmospheric CO_2_ and N_2_ pressures that are no higher than 0.1 bar and 2 bar, respectively. For humans, this CO_2_ tolerance limit is twice as high as previously suggested^14^. Such CO_2_ levels implicitly assume atmospheres containing roughly a standard amount of O_2_. At significantly lower O_2_ levels (e.g. <~0.13 bar), however, acclimatization to high CO_2_ levels does not seem possible^30^. Nevertheless, O_2_ can be ignored in the following HZ climate calculations for simplicity (see Methods). In contrast, the inner edge of the CLHZ is the same as that predicted by revised 1-D modeling results^7,31,32^. Thus (as customary), a 1-bar N_2_ classical HZ inner edge atmosphere pressure is also assumed for the CLHZ inner edge. Likewise, the modeled outer edge of the classical HZ (also with 1-bar N_2_) is determined by the distance beyond which the CO_2_ greenhouse effect is maximum^1^.

It is reasonable to ask if the above respiratory limits are applicable to complex life on Earth throughout its entire history (the Phanerozoic, corresponding to the past ~540 Myr). However, the answer to this question is likely “yes.” Phanerozoic CO_2_ levels were never higher than ~0.01 bar (< ~ 1/10 of the CO_2_ limit) whereas corresponding N_2_ pressures were comparable to that of the present day^33,34^. Thus, terrestrial complex life has always lived in atmospheres under N_2_ and CO_2_ pressures that have never exceeded these respiratory limits, validating the CLHZ as a tool that can be used in the search for Earth-like complex life on other planets.

## Results

**Figure 2:**
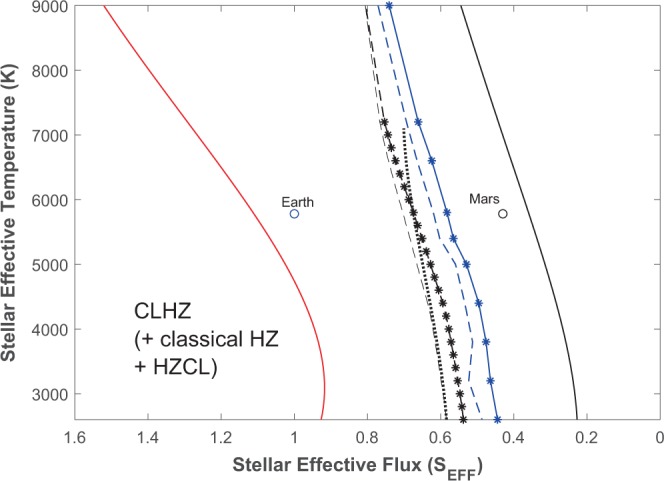
The Complex Life Habitable Zone (CLHZ) for A – M stars (2,600–9,000 K) compared to other definitions. The radiative-convective climate modeling CLHZ estimates (black curves) are shown alongside those of the EBM (blue curves) for both 1-bar (dashed curves) and 2-bar (curves with asterisks) N_2_ background atmospheres. All CLHZ outer edge limits assume a 0.1 bar atmospheric CO_2_ pressure. The inner (red curve) and outer (plain black curve) edges of the modeled classical HZ are also shown along with the 0.1 bar CO_2_ Habitable Zone for Complex Life (HZCL) (black dotted) of Schwieterman *et al*.^14^. The Earth and Mars around the Sun (5800 K) are shown for comparison.

The CLHZ is a subregion within the classical HZ and is calculated assuming 1-bar and 2-bar N_2_ background pressures (Fig. 2). The CLHZ has been calculated using two models (see Methods). The first involves the radiative-convective (RC) model of Ramirez and Kaltenegger^6,7^, which is similar to previous ones used to compute the classical HZ^1,3,14^. In addition, I revise this calculation with an updated version of an advanced energy balance model^35^ (EBM) (Fig. 2). Both models yield almost identical classical HZ limits, in agreement with previous RC model studies^1–3^. This is because the atmosphere is so optically thick at both classical HZ edges that cloud absorption becomes less important, producing more agreement between the EBM and cloud-free RC model results . As I show below, however, the calculated CLHZ limits differ between the two models.

The distance (*d* in AU) for the various HZ limits is calculated by the following^1^:

$$d=\sqrt{L/{S}_{EFF}}$$

Here, *L* is the stellar luminosity and (S_*EFF*_) is the effective stellar flux received by the planet normalized to that received by the Earth at 1 AU. For 1-bar N_2_ and 2-bar N_2_ background pressures, the RC model predicts a CLHZ outer edge of ~1.19 and 1.21 AU, respectively. With an inner edge located at 0.95 AU^31^, this yields a CLHZ width of ~0.24–0.26 AU. In comparison, the EBM computes HZ outer edges of ~1.27 and 1.31 AU, respectively, or a CLHZ width of 0.32–0.36 AU (~33–38% increase). For comparison, the EBM and RC model compute classical HZ limits of 0.95–1.67 AU (0.72 AU) in our solar system.

**Figure 3:**
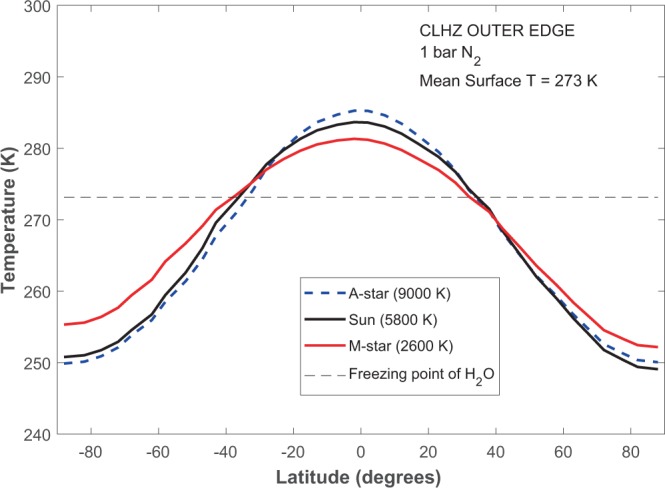
Latitudinal surface temperatures for planets near the outer edge of the CLHZ for representative A – M stars. Equator-pole temperature gradients are greatest for the A-star planet and least for the M-star planet.

**Figure 4:**
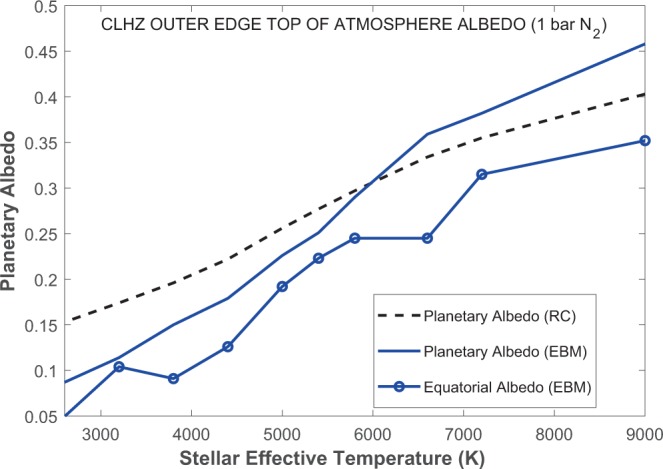
CLHZ outer edge planetary albedo calculated from radiative-convective climate (black dashed line) and energy balance (blue line) modeling simulations and equatorial albedo computed from energy balance (blue line with circles) modeling simulations for planets (with 1 bar N_2_) orbiting A – M stars (2,600–9000 K). Planetary albedo is lower in the EBM for stars cooler than ~ 6000 K. Although EBM planetary albedo is higher for planets orbiting hotter stars, equatorial regions still exhibit lower albedo values.

A wider CLHZ is predicted with the EBM for a few reasons. First, EBM equatorial temperatures can exceed the freezing point (273 K) even though mean surface temperatures do not (Fig. 3). In comparison, latitudinal temperature variations cannot be considered in the RC model computation, limiting calculations to a 273 K global mean surface temperature. Secondly, the RC model HZ calculations are calibrated such that cloud reflectivity for cooler planets near the outer edge of the CLHZ is similar to that of an Earth-like planet with a 288 K mean surface temperature^1,3^. However, this assumption overestimates the planetary albedo for cooler outer edge CLHZ planets (~273 K; Fig. 4), which are predicted to have lower cloud cover^36^. Thirdly, an Earth-like 30% land fraction is assumed in the EBM (see Methods), resulting in a somewhat weaker ice-albedo feedback than in the RC model aquaplanet, enabling effective heat transport at slightly larger orbital distances.

The CLHZ is slightly wider at the higher N_2_ pressure because of increased N_2_-N_2_ collision induced absorption and a decrease in the outgoing infrared flux, which more than offset an increase in planetary albedo (Fig. 2; see Methods). Up to now, I had assumed that complex life couldn’t evolve over time to withstand even higher N_2_ pressures. Interestingly, diving data suggest that some acclimatization to nitrogen narcosis might be possible, at least for a limited amount of time^29^. Motivated by this, I consider how our solar system’s HZ changes if we assume (for the moment) that complex life could evolve to breathe in a hypothetical 5-bar N_2_ atmosphere. For this sensitivity study, the RC model predicts that such worlds in our solar system can remain habitable at 1.24 AU (S_*EFF*_ = 0.65) whereas atmospheric collapse can be avoided as far as 1.36 AU (S_*EFF*_ = 0.54) in the EBM (nearly 60% classical HZ width). I find that the additional N_2_ opacity is sufficient to counter the ice-albedo feedback, allowing for effective planetary heat transfer even at relatively far distances. A similar result was found in Vladilo *et al*.^37^ at higher atmospheric pressures. This is not captured in the RC model because it has no latitudinal temperature distribution.

## Discussion and Conclusions

Although I find a CLHZ that is narrower than the classical HZ, it is significantly wider than recently predicted^14^ at the same CO_2_ level (0.1 bar), with an outer edge at 1.31 AU in our solar system (versus 1.19 AU with the RC model). The width of this CLHZ is more than 30% wider than previously computed for similar CO_2_ levels^14^, and nearly half that of the classical HZ (Fig. 2).

Schwieterman *et al*.^14^ argue that technological civilizations may not be possible on planets located near the outer edge of the classical HZ. However, these and similar calculations assume that complex life elsewhere would follow a similar evolutionary path as has the Earth. This need not be the case^38^. If complex life can evolve to breathe in even denser CO_2_ or N_2_ atmospheres, and acclimatization to narcosis is possible, as perhaps suggested by N_2_ diving data^29^, then the HZ for complex life may be larger than computed here, possibly rivaling the size of the classical HZ (see Results). Nevertheless, all respiratory limits become moot if they can be overcome through non-evolutionary means (e.g. genetic engineering) by a civilization that is even more advanced than ours.

Those caveats aside, if SETI efforts eventually prove successful, the CLHZ could be used to determine which HZ planets in a given system may harbor complex life. Otherwise, in the search for life in general (including simple life) we should employ the full extent of the HZ, ideally using the correct version of the HZ for the appropriate conditions^3,6,7,9^, which should be used in tandem with any potential biosignatures. The proper HZ to use for a given stellar system will be a function of variables, including stellar age^9,10,39^, and atmospheric composition^5–7^.

An ongoing uncertainty in calculated HZ limits is the location of the classical HZ inner edge for tidally-locked planets, particularly around M-dwarf stars. Some 3-D climate models predict that the inner edge for planets orbiting tidally-locked stars is somewhat closer to the star than what 1-D models predict^40–42^ suggesting a wider classical HZ and CLHZ. This is because a large substellar point cloud deck forms in these models, increasing the fraction of reflected starlight and causing the runaway greenhouse to be triggered at smaller orbital distances. However, the calculated limits differ widely among models and strongly depend on model assumptions, including convection and cloud schemes. More recent models have found better agreement with the 1-D limits^42^. Nevertheless, further improvement here requires a better understanding of clouds and convection in climate regimes that are far removed from Earth’s.

It was argued that atmospheric CO on mid to late M-dwarf HZ planets could accumulate sufficiently high to be lethal to complex life^14^. This may be possible on worlds that evolve Earth-like atmospheric conditions and chemistry. However, many M-dwarf HZ worlds that are currently located in the main-sequence HZ would have experienced lengthy runaway greenhouse episodes lasting hundreds of millions, if not more than a billion years. Such planets likely have their atmospheres eroded and surfaces desiccated^9,43^. Alternatively, some M-dwarf HZ planets may be ocean worlds that are very unlike our planet. In that case, complex life may be severely inhibited, if not impossible^35^.

To conclude, I define a complex life habitable zone (CLHZ) for A - M stars that is based on N_2_ and CO_2_ respiration limits for complex life on Earth, assuming that advanced life elsewhere will exhibit similar physiological limitations as humans and other air-breathing animals. This CLHZ applies both theoretical ideas and implications from experimental results. Although I find that the CLHZ is narrower than the classical HZ, it is wider than what was recently computed using a radiative-convective climate model^14^. Ultimately, progress on this problem requires improved observational and experimental analyses that continue to refine the constraints for complex life.

## Methods

### Planetary assumptions

The planet of interest is an Earth analogue, with the same mass, radius, and orbital period. Atmospheres are assumed to be fully-saturated. I use typical values for Earth’s obliquity (23.5 degrees) and a 70% ocean coverage for the EBM calculations. A 1- or 2-bar N_2_ atmosphere is assumed (unless otherwise indicated) for a 0.1 bar CO_2_ atmosphere. Oxygen is neglected because its absorption is very weak and has a negligible effect on HZ limits^1^. Ozone is also not included. See “*single column radiative-convective climate model*” sub-section for ozone discussion.

### Energy balance climate model

My advanced energy balance model (EBM) is an updated version of the non-grey latitudinally dependent model described in Ramirez and Levi^35^. The model assumes that planets in thermal equilibrium (on average) absorb as much energy from their stars as they emit. Planets are divided into 36 latitude bands that are each 5° wide. We assume the following radiative and dynamic energy balance equation for all latitude bands and time steps^44,45^.

1 $$C\frac{\partial T(x,t)}{\partial t}-\frac{\partial }{\partial x}D(1-{x}^{2})\frac{\partial T(x,t)}{\partial x}+OLR=S(1-A)$$

where, *x* is sine(latitude), *S* is the incoming stellar flux, *A* is the top of atmosphere albedo, *T* is surface temperature, *OLR* is the outgoing thermal infrared flux, *C* represents the overall ocean-atmospheric heat capacity, and *D* represents a calculated diffusion coefficient. A second order finite differencing scheme solves Eq. 1.

The EBM accesses lookup tables constructed by the radiative-convective climate model (described below) which contain interpolated radiative quantities (stratospheric temperature, outgoing longwave radiation, planetary albedo) as a function of zenith angle, stratospheric temperature, surface temperature, pCO_2_, pN_2_, and surface albedo, The current EBM operates over a parameter space spanning 10^−5^ bar <pCO_2_ < 35 bar, 1 < pN_2_ < 10 bar, 150 K < T < 390 K, for zenith angles from 0 to 90 degrees and surface albedo values ranging from 0 to 1.

The model distinguishes between land, ocean, ice, and clouds. As the atmosphere warms near and above the freezing point, water clouds form, with cloud coverage (*c*) dictated by Eq. 2:

2 $$c=\,\min (0.72\,\log ({F}_{C}/{F}_{E}+1),1)$$

Here, F_*C*_ is the convective heat flux whereas F_*E*_ is the convective heat flux for the Earth at 288 K (~90 W/m^2^) in our model. This equation is similar to that used in the CAM GCM^36,46^, and yields an Earth-like cloud cover of ~50% at a mean surface temperature 288 K. When temperatures become cold enough for CO_2_ clouds to form, we assume a constant cloud fraction of 50%, following GCM studies^47^. Nevertheless, CO_2_ cloud formation does not occur in CLHZ simulations. H_2_O cloud albedo is assumed to be a linear function of zenith angle (in radians)^37,48^, (Eq. 3):

3 $${a}_{c}=\alpha +\beta z$$

Here, *a*_*c*_ is cloud albedo, whereas the fitting constants α and β are −0.078 and 0.65, respectively.

Fresnel reflectance data for ocean reflectance at different zenith angles is used. Ice absorption is treated in UV/VIS and near-infrared channels and the proper contribution is calculated for each star type.

I implement the following albedo parameterization for snow/ice mixtures, similar to that in Curry *et al*.^49^ (Eq. 4)

$$\alpha (visible)=(\begin{array}{cc}0.7 & T\le 263.15\\ 0.7-0.020(T-263.15); & 263.15 < T < 273.15\\ 0.5 & T\ge 273.15\end{array})$$

4 $$\alpha (nir)=(\begin{array}{ll}0.5 & T\le 263.15\\ 0.5-0.028(T-263.15); & 263.15 < T < 273.15\\ 0.22 & T\ge 273.15\end{array})$$

The EBM also models water ice coverage within a latitude band (*fice*) to temperature based on empirical data^50^. I find the following fit (Eq. 5):

5 $$fice=(\begin{array}{ll}1. & ,\,T\le 239\\ 1-\exp ((T-273.15)/12.5) & ,\,239 < T < 273.15\\ 0 & ,\,T\ge 273.15\end{array})$$

Zonally-averaged, surface albedo at each latitude band is calculated via the following (Eq. 6):

6 $${a}_{s}=(1-fc)\{(1-{f}_{o}){a}_{l}+{f}_{o}[{f}_{i}{a}_{i}+(1-{f}_{i}){a}_{o}]\}+{f}_{c}{a}_{c}$$

Here, *a*_*s*,_ *a*_*c*,_ *a*_*o*_, *a*_*i*_*, and a*_*l*_ are the surface, cloud, ocean, ice, and land albedo, respectively. Likewise, *f*_*c*_, *f*_*o*_, and *f*_*i*_ are the cloud, ocean, and ice fraction, respectively. Following Fairen *et al*.^51^, at sub-freezing temperatures, the maximum value between ice and cloud albedo is chosen to prevent clouds from artificially darkening a bright ice-covered surface.

Heat transfer efficiency is determined by the following parameterization (Eq. 7):

7 $$D={D}_{o}\left(\frac{p}{{p}_{o}}\right)\left(\frac{{c}_{p}}{{c}_{p,o}}\right){\left(\frac{{m}_{o}}{m}\right)}^{2}{\left(\frac{{\Omega }_{o}}{\Omega }\right)}^{2}$$

Here, values with the “*o*” subscript indicate Earth’s values. *c*_*p*_ is the heat capacity, *m* is atmospheric molecular mass, *p* is atmospheric pressure,  *Ω* is rotation rate, *D* is the globally-averaged heating efficiency, with a value of *D*_*o*_ = 0.58 Wm^−2^K^−1^ for the Earth^35,48^.

### The single column radiative-convective climate model

The single-column radiative-convective climate model^6,7^, has 55 thermal infrared and 38 solar wavelengths. Model atmospheres are composed of 100 vertical logarithmically-spaced layers that reach ~5 × 10^−5^ bar at the top of the atmosphere. In warmer atmospheres, water vapor convects and the model relaxes to the moist adiabatic value if exceeded. Likewise, when atmospheres are cold enough for CO_2_ to condense, lapse rates are adjusted to the CO_2_ adiabat^1^. The atmosphere expands when temperatures are warm enough although this does not have much of an effect in these relatively cool simulations (<300 K)^52,53^.

The radiative-convective climate model utilizes HITRAN^54^ at lower temperatures (<300 K) and HITEMP^55^ at higher temperatures for water vapor and HITRAN for CO_2_. Far wing absorption in the CO_2_ 15-micron band utilizes the 4.3 micron region as a proxy^56^. The BPS water vapor continuum is overlain between 0 and 19,000 cm^−1^ ^57^. I implement CO_2_-CO_2_ and N_2_-N_2_ CIA^58–61^ A standard Thekeakara solar spectrum^62^ is applied to the Sun whereas the spectra for the remaining A – M stars (T_EFF_ = 2,600–9000 K) are modeled with Bt-Settl data^63^. One solar zenith angle at 60 degrees is assumed for the radiative-convective climate model habitable zone calculations.

As is typical for habitable zone calculations, I assume a temperature profile and calculate the fluxes that support it in what are called inverse calculations^1^. I prescribe stratospheric and mean surface temperatures of 160 K and 273 K, respectively. Results are relatively insensitive to ~30–40 K differences in stratospheric temperature^1^. The surface albedo (0.31 in my model) is a tuning parameter which gives the correct temperature structure for the Earth. Unlike the real Earth, neither oxygen nor ozone are present in these calculations. Ozone absorption cannot be reliably calculated with inverse calculations because ozone produces temperature inversions that are not captured by assuming a constant temperature stratosphere^7^. Nevertheless, the difference from making this assumption is no more than a couple of degrees in mean surface temperature^1^.

## Supplementary information

Supplementary information.

## Data Availability

All data from this study are included in this publication.
