# Supplementary material for: A Complex Life Habitable Zone Based On Lipid Solubility Theory
Source: Sci Rep. 2020 May 4;10:7432. doi: 10.1038/s41598-020-64436-z (PMC7198600; doi:10.1038/s41598-020-64436-z)
Supplement: Supplementary file 1 — Supplementary information. [file 41598_2020_64436_MOESM1_ESM.pdf]

SUPPLEMENTARY INFORMATION TO:

A COMPLEX LIFE HABITABLE ZONE BASED ON LIPID SOLUBILITY THEORY

Ramses M. Ramirez[1,2]

[1]Earth-Life Science Institute, Tokyo Institute of Technology, Tokyo, Japan

[2]Space Science Institute, Boulder, Co, USA

More Experimental Verification of Derived Respiratory Limits for CO_2

As discussed in the main text, many studies have been conducted to assess the acclimatization potential of animals to high CO_2 doses (i.e., hypercapnia) (e.g., Stinson et al. 1970; Stinson et al. 1971; Kantores et al. 2006; Ryu et al. 2010). Although there has been limited experimentation on humans, these are fewer in number (e.g., Schaefer et al. 1963; Storm and Giannetta 1974), likely due to ethical concerns. Here, I clarify a few points and cite additional studies that lend further support to the ~0.1 bar CO_2 upper respiratory limits for humans and animals that were derived from the Meyer-Overton correlation (Meyer et al. 1899; 1901; Overton et al. 1901). Admittedly, there are caveats and limitations to this correlation, including it to be a poor fit for complex perfluorinated compounds and a debate as to whether anesthetic action occurs in lipid bilayers or protein binding sites. It could be that lipid bilayers are a necessary, but insufficient condition for anesthetic action (see review by Franks 2008 and references therein). Nevertheless, as explained in the main text, the correlation works well for the specific cases of CO_2 and N_2 given their reasonable agreement with the correlation (Figure 1 in main text) along with consistency with several decades of CO_2 and N_2 respiratory experiments in humans and animals. I discuss some of these experiments in greater detail, including their results and implications below.

Acclimatization versus Acute Exposures

I first illustrate the difference between gradual acclimatization and acute exposures to high CO_2 levels. The latter greatly increases the risk of mortality whereas the former allows the body to tolerate higher CO_2 levels than what would be possible through acute exposure. Although adult rhesus monkeys have acclimated with no problems to 0.06 bar CO_2, with adaptation to even higher levels possible (Stinson 1970), acute exposures of 30% CO_2 per hour after a 3-day acclimatization period killed similar monkeys in a later study (Stinson 1971). This is not surprising. The magnitude (0.3 bar CO_2 is far above the CO_2 threshold here) of such a sudden CO_2 increase made it impossible for further acclimatization.

In contrast, CO_2 levels were gradually incremented in a stepwise fashion (0.02, 0.04, 0.08, 0.12, and 0.16 bar) in the adult sheep experiment (Hoover et al. 1970;1971). In addition, after attaining the specified CO_2 level, it was held there for 7 subsequent days, further providing for a smooth acclimatization process. The one exception was the 0.16 bar CO_2 level, in which the duration was reduced to 5 days because of the resultant negative stresses (Hoover 1971). Therefore, these experiments suggest a CO_2 limit between ~0.12 and 0.15 bar, which is consistent with the 0.15 bar CO_2 limit derived in the text for adult animals.

High CO_2 is harmful under low O_2

As mentioned in the text, the ability to adapt to high CO_2 levels (>0.05 bar) diminishes at low oxygen levels (hypoxia) (Kantores et al. 2006). This likely explains why birth defects were found in newborn rats exposed to high (0.06 bar) CO_2 and low (0.1 bar) O_2 (Haring 1966). However, newborn rats under ambient oxygen levels could adapt to even higher CO_2 levels (~0.07 -0.09 bar) (e.g. Kantores et al. 2006; Ryu et al. 2010). Increased CO_2 actually helps transport O_2 to the blood, in combination with moderately high (>~0.13 bar) O_2 levels (e.g. Kantores et al. 2006), which partially explains why adaptable hypercapnia can be beneficial. However, this benefit is reduced at lower O_2 levels (~ < 0.13 bar), and the maximum respiration limit of CO_2 also decreases (e.g. Kantores et al. 2006). Again, this analysis is consistent with my inferred 0.1 bar upper respiratory limit for CO_2 for standard oxygenated conditions.

Medical benefits to increased CO_2

In fact, hypercapnia appears to increase lung volume and development in newborn rats, again pointing to a beneficial role at high CO_2 levels (Ryu et al. 2010). Indeed, therapeutic (or permissive) hypercapnia in humans provides positive stresses to the brain and organs that make it possible to treat certain ailments (e.g. Laffey et al. 2000; Ambalavanan et al. 2001). Although much more experimental work must be conducted, including for even longer durations (e.g., months, years), the literature suggests that humans and animals have a much higher acclimatization potential to elevated CO_2 levels than perhaps has been realized.

Applicability to non-mammals

The experiments discussed in this paper suggest that the Meyer-Overton relation and derived respiratory limits are consistent across mammalian vertebrates. However, the Meyer-Overton correlation can describe non-mammalian respiration as well. For instance, some experiments using other anesthetic gases (halothane, isoflurane) suggest close correspondence among birds, fish, and mammals (e.g., Zalucki and Van Swinderen 2016). Experiments with insects and nematodes (e.g., worms) also show strict adherence to the Meyer-Overton correlation although some nematodes apparently tolerate much higher doses than do other animals (e.g., Allada and Nash, 1993; Wasserkort and Koller, 1997; Kayser et al. 1998). However, this tiny (~ 1mm long) nematode (*C. elegens*) is among the simplest of complex organisms, possessing a very rudimentary nervous system and no brain (Zalucki and Van Swinderen 2016). Thus, the CO_2 and N_2 limits discussed in this paper may not be applicable to very simple forms of complex life.

REFERENCES:

Allada, R. & Nash, H.A. Drosophila melanogaster as a model for study of general anesthesia: the quantitative response to clinical anesthetics and alkanes. *Anesthesia and analgesia*, **77**,1, pp.19-26 (1993).

Ambalavanan, N. & Carlo, W.A. Hypocapnia and hypercapnia in respiratory management of newborn infants. *Clinics in perinatology*. **28**, 3, 517-531 (2001).

Franks, N.P. General anaesthesia: from molecular targets to neuronal pathways of sleep and arousal. *Nature Reviews Neuroscience,* **9**, 5, 370-386 (2008).

Haring, O.M., 1966. Cardiac malformations in the rat induced by maternal hypercapnia with hypoxia. *Circulation research*, **19**, 3, 544-551 (1966).

Hoover, W. H., Young, P. J., Sawyer, M.S., & Apgar, W. P. Ovine physiological responses to elevatied ambient carbon dioxide. *J. Appl. Physiol.* **29**, 32–35 (1970).

Hoover, W.H., Sawyer, M.S. and Apgar, W.P., 1971. Ovine nutritional responses to elevated

ambient carbon dioxide. *The Journal of nutrition*, **101**, 12, 1595-1600.

Kantores, C., McNamara, P.J., Teixeira, L., Engelberts, D., Murthy, P., Kavanagh, B.P. and Jankov, R. . Therapeutic hypercapnia prevents chronic hypoxia-induced pulmonary hypertension in the newborn rat. *Am. J. Physiol. Cell. Mol. Physiol.* **291**, L912–L922 (2006).

Kayser, B., Rajaram, S., Thomas, S., Morgan, P.G. and Sedensky, M.M. Control of anesthetic response in C. elegans. *Toxicology letters*, **100,** pp.339-346 (1998).

Knowlton, P. H., Hoover, W. . & Poulton, B. R. Effects of high carbon dioxide levels on the nutrition of sheep. *J. Anim. Sci.* **28**, 554–556 (1969).

Laffey, J.G., Tanaka, M., Engelberts, D., Luo, X., Yuan, S., Keith Tanswell, A., Post, M., Lindsay, T. & Kavanagh, B.P. Therapeutic hypercapnia reduces pulmonary and systemic injury following in vivo lung reperfusion. *American journal of respiratory and critical care medicine*, **162**, 6, 2287-2294 (2000).

Meyer, H. Zur Theorie der Alkoholnarkose. *Naunyn. Schmiedebergs. Arch. Pharmacol.* **42**, 109–119 (1899).

Meyer, H. Zur Theorie der Alkoholnarkose. *Arch. für Exp. Pathol. und Pharmakologie* **46**, 338–346 (1901).

Overton, C. Studien über die Narkose zugleich ein Beitrag zur allgemeinen Pharmakologie. *Gustav Fischer, Jena, Switz.* (1901).

Ryu, J., Heldt, G.P., Nguyen, M., Gavrialov, O. and Haddad, G.G. Chronic hypercapnia alters lung matrix composition in mouse pups. *Journal of Applied Physiology*, **109**, 1, 203-210 (2010).

Schaefer, K.E., Hastings, B.J., Carey, C.R. & Nichols Jr, G. Respiratory acclimatization to carbon dioxide. *Journal of applied physiology*, **18**, 6, 1071-1078 (1963).

Stinson, J. M. & Mattsson, J. L. Tolerance of Rhesus Monkeys to Graded Increase in Environmental CO_2-Serial Changes in Heart Rate and Cardiac Rhythm. *Aerosp. Med.* **41**, 415–418 (1970).

Stinson, J.M., Smith, H.P., Smith, J.H. and Rumsey, A.N. Carbon Dioxide Tolerance After Adaptation to Hypercarbia. *Archives of Environmental Health: An International Journal*, **22**, 4, 440-443 (1971).

Storm, W. F. & Giannetta, C. L. Effects of hypercapnia and bedrest on psychomotor performance. *Aviat. Space. Environ. Med.* **45**, 431–433 (1974).

Wasserkort, R. and Koller, T., March. Screening toxic effects of volatile organic compounds using Drosophila melanogaster. In *Journal of Applied Toxicology: An*

International Forum Devoted to Research and Methods Emphasizing Direct Clinical, Industrial and Environmental Applications (Vol. 17, No. **2**, pp. 119-125). Chichester: John Wiley & Sons, Ltd (1997).

Zalucki, O. and Van Swinderen, B. What is unconsciousness in a fly or a worm? A review of general anesthesia in different animal models. *Consciousness and cognition,* **44**, pp.72-88 (2016).
